# Efficacy of Self-Management Smartphone-Based Apps for Post-traumatic Stress Disorder Symptoms: A Systematic Review and Meta-Analysis

**DOI:** 10.3389/fnins.2020.00003

**Published:** 2020-01-24

**Authors:** Andreas Goreis, Anna Felnhofer, Johanna Xenia Kafka, Thomas Probst, Oswald D. Kothgassner

**Affiliations:** ^1^Department for Clinical and Health Psychology, Faculty of Psychology, University of Vienna, Vienna, Austria; ^2^Outpatient Unit for Research, Teaching and Practice, Faculty of Psychology, University of Vienna, Vienna, Austria; ^3^Department of Pediatrics and Adolescent Medicine, Medical University of Vienna, Vienna, Austria; ^4^Department of Child and Adolescent Psychiatry, Medical University of Vienna, Vienna, Austria; ^5^Department for Psychotherapy and Biopsychosocial Health, Danube University Krems, Krems, Austria

**Keywords:** smartphone app, PTSD, post-traumatic stress disorder, mHealth, trauma intervention, depression, meta-analysis, mobile phone intervention

## Abstract

Post-traumatic stress disorder (PTSD) symptoms are prevalent in both civilian and military service members. As the number of smartphone-based applications (apps) grows rapidly in health care, apps are also increasingly used to help individuals with subthreshold PTSD or full PTSD. Yet, if the apps are self-managed, the feasibility and efficacy of such interventions are still rather unclear in these two populations with PTSD symptoms. Hence, the present meta-analysis set out to evaluate the effect of self-management smartphone-based apps on PTSD and depressive symptoms in populations with subthreshold PTSD or full PTSD. Studies were included if they conducted randomized controlled trials or pre-post comparisons. Six studies (*n* = 2 randomized controlled trials) were identified for meta-analysis. In pre-post comparisons, *N* = 209 participants were included in the analyses. In randomized controlled trials, *N* = 87 participants received smartphone-based self-management interventions and *N* = 82 participants were in waitlist control conditions. Meta-analysis for pre-post comparisons concluded an effect of *g* = 0.55 (*p* < 0.001) regarding the overall reduction in PTSD symptoms (*n* = 6) and *g* = 0.45 (*p* < 0.001) for reduction in depressive symptoms (*n* = 5). Yet, in randomized controlled trials, no significant difference was found between app-based treatment and waitlist control groups (*g* = 0.09, *p* = 0.574). The duration of the interventions did not significantly influence the results. Overall, despite positive pre-post effects, current results indicate that smartphone-apps for PTSD patients are not significantly more effective than waitlist control conditions. Nevertheless, a combined smartphone and standard therapy approach may be a fruitful field for future research.

## Introduction

Post-traumatic stress disorder (PTSD) is a cause of substantial disability in both civilian and military populations, leading to long-term problems for individuals, families, and society in terms of compromised emotional well-being, productivity loss, and high cost of treatment (Kessler, 2000; Breslau et al., 2004; Buckley et al., 2004; Cohen et al., 2009; Kok et al., 2012; Marmar et al., 2015). PTSD is characterized by a multitude of symptoms resulting from exposure to one or more traumatic events (World Health Organization, 1993; American Psychiatric Association, 2013). Individuals with PTSD are typically affected by anhedonia, emotional numbness, social detachment, unresponsiveness to external stimuli, insomnia, and suffer from hyperarousal (Elhai and Palmieri, 2011; e.g., Armour et al., 2016). The experience of traumatic events is also associated with elevated symptoms of depression and anxiety (Etkin and Wager, 2007; Mandelli et al., 2015).

PTSD has an estimated lifetime prevalence ranging from 2% in Europe (Darves-Bornoz et al., 2008; Maercker et al., 2008) to 7% in the United States (Kessler et al., 2005), and 4% in a cross-national study of 24 countries (Koenen et al., 2017). Furthermore, a significant number of individuals experience symptoms of subthreshold or subclinical PTSD in response to traumatic events that do not meet diagnostic criteria (Brancu et al., 2016). Subthreshold PTSD has previously been identified to affect around 20% of U.S. veterans returning from Afghanistan (Hoge et al., 2006) and prevalence estimates for civilian populations are mirroring—at least—the prevalence rates of those with full PTSD (Stein et al., 1997; Marshall et al., 2001; Breslau et al., 2004; Bergman et al., 2015). Research has shown that levels of distress and functional impairment are significantly heightened for individuals with subthreshold PTSD (e.g., Mylle and Maes, 2004), underscoring the fact that both subthreshold PTSD and the full PTSD cause impairment and represent considerable public health concerns (Bergman et al., 2015).

Evidence-based treatments are available for PTSD (Foa et al., 2009), and guidelines generally recommend exposure therapy and cognitive therapies, and pharmacological treatment as an adjunct treatment (for an overview of psychological treatments see Cusack et al., 2016). Literature on treatment options for subthreshold PTSD is limited (Dickstein et al., 2013), but treatment with lower intensity may be favorable (Shiner et al., 2012; Korte et al., 2016). Many affected individuals, however, remain without treatment due to negative beliefs about efficacy, stigma, logistic reasons, or shortage of qualified treatment centers in the adjacent geographic region (Hoge et al., 2004; Shalev et al., 2011; Kazdin and Rabbitt, 2013). Early and accessible interventions are equally important in subthreshold PTSD, as 25% of those affected develop the full PTSD (Marshall et al., 2001; Breslau et al., 2004; Cukor et al., 2010).

Innovative technology, such as applications (apps) for smartphones, can address the need for accessible and effective interventions after traumatic experiences, especially on a population level (Cernvall et al., 2018). Smartphones are carried by a majority of adults with ownership rates ranging between 77% in the U.S. (Pew Research Center, 2018) and 79% in the European Union (Eurostat, 2016). Promisingly, no ethnic disparities exist in smartphone ownership in U.S. adults (Pew Research Center, 2018) and applications for smartphones could be a feasible means of reaching minority populations with a possibly limited access to health care (López et al., 2012). Applications allow individuals to approach specific treatments at their own pace, individually, and confidentially, which may result in greater acceptance and compliance (Juarascio et al., 2014). Emerging evidence suggests that smartphone applications improve depression and anxiety symptoms (Donker et al., 2013; Firth et al., 2017), health behaviors such as physical activity, diet (Schoeppe et al., 2016), smoking cessation (Whittaker et al., 2016), and reduces alcohol consumption (Gustafson et al., 2014). Preliminary results also exist for potential benefits in patients with schizophrenia (Firth and Torous, 2015) and eating disorders (Juarascio et al., 2014).

Based on this, a multitude of applications, which specifically target subthreshold PTSD have been developed. In a literature review of mobile health apps for PTSD, Rodriguez-Paras et al. (2017) found 45 publicly available PTSD-specific apps in their recent review and they stated that minimal effort and transparency has been made regarding development, usability, and validation of this plethora of apps. The PTSD Coach app, for example, was jointly developed by the U.S. Department of Veterans Affairs' and the Department of Defense, providing users with self-management, psychoeducative elements concerning PTSD symptoms and treatment, symptom monitoring, and coping skills (U. S. Department of Veterans Affairs, 2011a,b; Possemato et al., 2016). PTSD Coach is available for iOS and Android devices, and preliminary studies reported a high satisfaction and acceptance among veteran (Kuhn et al., 2014) and community samples (Miner et al., 2016). Another app, PE Coach (U. S. Department of Veterans Affairs, 2017a,b), was also developed by the U.S. Department of Veteran Affairs and provides psychoeducation, symptom tracking, and—optionally—support features to improve patient compliance (e.g., appointment reminders, audio recordings, imaginal exposure homework). It was previously utilized to support users who were in primary care settings or receiving therapy (Reger et al., 2013, 2015). Some studies have been conducted to test the efficacy of these applications for individuals with (subthreshold) PTSD (Miner et al., 2016; Possemato et al., 2016; e.g., Kuhn et al., 2017). Results were promising, with moderate to large effects (*d* = 0.78) regarding the reduction of PTSD-symptoms post-intervention in the PTSD Coach group when compared to a waitlist-condition (Miner et al., 2016). In another study, 57% of PTSD Coach users reported a reduction of PTSD symptoms compared to 26% in a waitlist condition (Kuhn et al., 2017). In both studies, however, the two groups did not differ significantly in PTSD or depressive symptoms post treatment. Yet, sample sizes for the PTSD Coach condition were small in both studies (*n* = 25 in Miner et al., 2016; *n* = 62 in Kuhn et al., 2017), possibly impeding significant differences to be detected. Similar patterns emerged in Cernvall et al. (2018) with 11 participants, pre-post effect sizes for the reduction of symptoms were moderate for PTSD and depressive symptoms (*d* = 0.51 and *d* = 0.58, respectively), but both failed to reach nominal significance. As symptoms of depression and anxiety often have profound effects on affected individuals that overlap and co-occur with PTSD symptoms (Norris et al., 1997; e.g., Luxton et al., 2010) it is of additional interest to investigate the efficacy of smartphone-based apps on depressive and anxiety symptoms.

It is discernable that this field of research is underpowered and conclusions about the benefits of smartphone-based applications cannot be drawn on single trials alone. A recent study (Wickersham et al., 2019) reviewed the efficacy of mobile interventions, both self-managed and with clinician support, for the treatment of PTSD symptoms in randomized controlled trials (RCTs) and found inconclusive yet promising results, with a decrease of symptoms in app-based treatments, but not compared to control groups. To evaluate the efficacy of self-managed apps alone, further granulation and meta-analysis of individual studies is needed. We therefore present a meta-analysis on all available studies assessing the effects of self-management smartphone-based applications for PTSD treatment. The aim of the present meta-analysis is two-fold: (1) to conduct a meta-analysis of studies reporting the effect of self-managed mobile application on PTSD symptoms, and (2) to conduct a meta-analysis of studies reporting the effect of mobile applications on depression and anxiety symptoms as secondary outcome variables.

## Method

### Search Strategy and Inclusion Criteria

A search of MEDLINE, Scopus, and Web of Science was conducted using the keywords “PTSD OR trauma OR posttraumatic-stress disorder AND Smartphone OR App OR Application OR mobile phone” from the beginning of database records until January 2019. Studies were eligible to be included in the meta-analysis if they (i) conducted randomized controlled trials with waitlist controls or (ii) pre-post studies assessing the effect of self-management smartphone-based apps on PTSD symptoms. No other inclusion or exclusion criteria were applied. No limitations on language or publication status were invoked. We additionally coded and analyzed symptoms of depression and anxiety if they were reported. Furthermore, Google Scholar alerts were enabled to ensure inclusion of accepted articles and articles in preprint, and authors were contacted to ensure inclusion of unpublished studies. Two Authors (ODK and JXK) independently examined the title, abstract, and main text of each study and full text papers were obtained where necessary to evaluate inclusion. Any discrepancies were discussed by the two authors. Final inclusion was based on the following criteria:

1. Participants: Individuals with varying severity of PTSD symptoms as indicated by self-report questionnaires or via clinical interview conducted by a psychologist or physician.
2. Intervention: Self-managed smartphone-based apps.
3. Comparison: Studies with and without control groups were included.
4. Outcomes: Reported at least a PTSD symptom severity score before and after the intervention.
5. Study design: Pre-post studies or randomized controlled trials.

**Figure 1:**
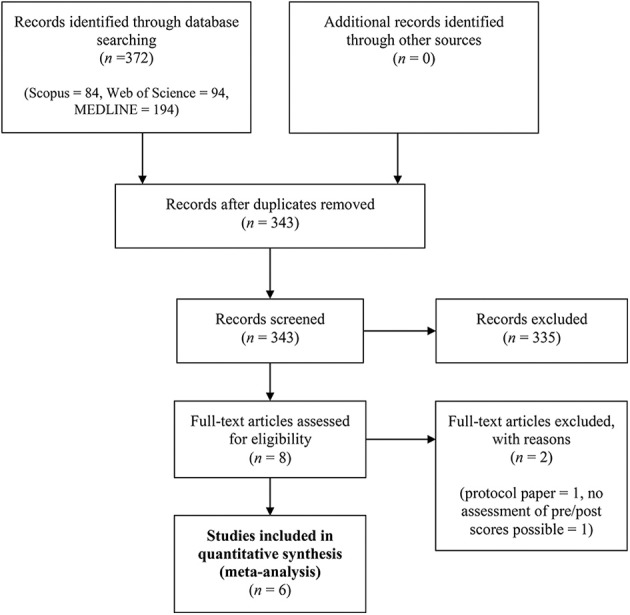
PRISMA flowchart of screening, exclusion, and inclusion criteria.

Exclusion of documents occurred at each stage (see Figure 1 for PRISMA flow diagram and Supplementary Table 3 for PRISMA checklist). The initial search generated 343 results. After the article selection process, six studies were identified and included in our meta-analysis.

### Data Extraction and Analysis

To analyze the effect of app-based interventions from pre to post, we computed the standardized mean difference (Hedges' *g*) of PTSD-symptoms, depressive symptoms, and anxiety symptoms based on means and standard deviations (Dunlap et al., 1996) before and after the app-based intervention. We used the formula *d* = (*M*_pre_ − *M*_post_)/*SD*_pooled_, where M_pre_ is the mean of the measure before the intervention and *M*_post_ after the intervention, with *SD*_pooled_ as the standard deviation for both measurements, defined as *SD*_pooled_ = SQRT(${SD}_{\text{pre}}^{2}$ + ${SD}_{\text{post}}^{2}$)/2 (Lakens, 2013). For the standardized mean difference between intervention and control groups as indicator of the efficacy of the intervention in randomized-controlled trials, we calculated Cohen's *d* for the post-intervention scores, based on means and standard deviations, with the formula *d* = (*M*_Intervention_ –*M*_Control_)/*SD*_pooled_, with the respective means of measurements for the intervention and control groups. To investigate changes from baseline separately in the intervention and control groups of the RCTs, we also computed the above-mentioned effect sizes for pre-post changes. Means, standard deviations and sample sizes were retrieved and entered into a spreadsheet. The calculations of the effect sizes and the subsequent meta-analysis were then conducted using the package metafor for R (Viechtbauer, 2010), which automatically corrects Cohen's *d* for a potential positive bias in small samples, yielded the effect size Hedges' *g* (Hedges, 1981). Following general convention (Cohen, 1988), an effect size of 0.20 was considered a small effect, 0.50 a moderate effect, and 0.80 a large effect. Random effects models were applied to estimate aggregated effect sizes (Borenstein et al., 2011). Heterogeneity across study outcomes was reported with *I*^2^ values, where 0 to 40% might not be important, 30 to 60% may represent moderate heterogeneity, and 50 to 90% may represent substantial heterogeneity (Higgins and Green, 2011).

Egger's regressions were conducted to analyze indications for publication bias (Sterne and Egger, 2005). Trim-and-fill analyses were calculated to provide estimates for adjusted effect sizes and, based on funnel plot asymmetry, numbers of imputed missing studies (Duval and Tweedie, 2000). Publication bias can be tested by entering data in a funnel graph (a plot of dispersion between study effect and a measure of study size). A symmetrical inverted distribution of the studies around the mean effect size represented in the funnel would indicate an absence of publication bias. Moderator analysis (meta-regression) was calculated to test whether the durations of interventions (in weeks) moderate the effect of the self-management app-based interventions on PTSD and depressive symptoms. The alpha level was set at 5% for all analyses. All data and codes are stored on a repository of the Open Science Framework (doi: 10.17605/OSF.IO/DZJT7).

### Risk of Bias Assessment

We assessed risk of bias for each study using predefined criteria based on the AHRQ Method Guide for Comparative Effectiveness Reviews (Viswanathan et al., 2018). Therefore, categories regarding randomization, selection and attrition bias, confounding bias, measurement bias and statistical problems were included for coding. We rated all studies according to low, moderate or high risk of bias. Results assessed as having low risk of bias are considered to be valid, moderate risk of bias indicate some risk of bias, but probably this does not invalidate its results, a high risk indicates significant issues with design, measurement, conduct or analysis, all of which probably invalidates the results. We predefined that inappropriate methods of randomization, no control for confounding factors high attrition ≥40% or differential loss ≥30%, problems in participant selection and adequate statistical power are reasons for high risk of bias ratings. However, we rated grades of overall strength of evidence (SOE) according to Owens et al. (2010) for all studies as displayed in Table 1. The supplemental materials (Supplementary Tables 1, 2) deliver an overview concerning the coding categories and risk of bias assessments. The assessments were independently determined by two investigators (AG and ODK); disagreements between the two investigators were discussed.

**Table 1:** Characteristics of the six studies included in the meta-analysis.

**Study**	**Country**	**Sample**	**Treatment Group**			Control Group^*^			**App**	**Duration**	**Design**	**SOE**
			***n***	**Age *M* (*SD*)**	**% male**	***n***	**Age *M* (*SD*)**	**% male**				
Cernvall et al. (2018)	Sweden	General population with full or partial PTSD (according to CAPS-5)	11	38.6 (Range 32–55)	27	–	–	–	PTSD Coach	4 weeks	Pre-test post-test design	low
Kuhn et al. (2017)	USA	General population with PCL-C score > 34 (subthreshold)	62	39.43 (15.16)	26	58	39.12 (14.08)	36	PTSD Coach	3 months	RCT with waitlist control group	high
Miner et al. (2016)	USA	General population with PCL-C score > 24 (subthreshold)	25	whole sample: 45.7 (13.9)	16	24	–	21	PTSD Coach	1 month	RCT with waitlist control group	moderate
Possemato et al. (2016)	USA	Veterans with PCL-S score > 40 (subthreshold)	10	42 (12)	95	–	–	–	PTSD Coach	2 months	RCT with clinician-support control group	low
Roy et al. (2017)	USA	Military service members and relatives with PCL score > 27 (subthreshold)	72	33.97 (10.8)	50	–	–	–	LifeArmor, PE Coach, Eventful, Positive Activity Jackpot, Tactical Breather, Daily Yoga, Simple Yoga	6 weeks	RCT with clinician-support control group	moderate
Tiet et al. (2019)	USA	Military service members with PC-PTSD score > 2 (probable PTSD)	29	Median: 61	97	–	–	–	PTSD Coach	4 months	Pre-test post-test design	moderate

The description of Tiet et al. (2019) is the treatment arm without clinician support. SOE, Strength of Evidence; CAPS-5, Clinician-Administered PTSD for DSM-5 (Weathers et al., 2017). PCL, PTSD Checklist; PC-PTSD, Primary Care-PTSD Screen (Prins et al., 2004).

* *Only RCTs with waitlist control groups are reported*.

## Results

### Study Characteristics

The six studies included in our meta-analysis covered data from 209 participants in self-management app-based intervention groups and 82 in control groups. All study samples included persons with both PTSD and subthreshold PTSD. Three studies (Possemato et al., 2016; Roy et al., 2017; Tiet et al., 2019) included samples of military service members, the remainder evaluated participants from the general population. All studies were conducted in the U.S., with the exception of Cernvall et al. (2018), which was conducted in Sweden. Additionally, all studies used the same application (PTSD Coach, U. S. Department of Veterans Affairs, 2011a,b), with the exception of Roy et al. (2017), who provided their sample with a multitude of applications with varying content (e.g., LifeArmor and PE Coach for psychoeducation concerning prolonged exposure, Tactical Breather for breathing exercises, Eventful to facilitate positive social engagement). See Table 1 for detailed study characteristics and SOE assessments for each study.

Four of these six studies were included as pre-post comparisons (Possemato et al., 2016; Roy et al., 2017; Cernvall et al., 2018; Tiet et al., 2019) and two were included as randomized controlled trials with waitlist control conditions (Miner et al., 2016; Kuhn et al., 2017). Two studies (Possemato et al., 2016; Roy et al., 2017) had a randomized controlled design, but only pre-post comparisons were included to be in line with the aim of the present meta-analysis, i.e., to examine the effect of self-management apps. One study (Possemato et al., 2016) randomly assigned participants to either self-managed or clinician-managed PTSD Coach conditions (*n* = 10 per condition). The clinician managed condition received four 20-min sessions (via phone) which focused on providing instructions for app use, setting goals for symptom reduction, and assigning activities between sessions (Possemato et al., 2016). In order to assure cross-study comparability, we only included the self-managed PTSD Coach condition in which no support by a clinician was provided in our meta-analysis as a pre-post comparison. Roy et al. (2017) compared the efficacy of an app-based intervention supported by daily brief text messages with elements of resilience enhancement and cognitive-behavioral therapy to a self-management control group without such support. As the aim of the present meta-analysis was to evaluate the effect of self-management app-based interventions, we included only the self-management group of the study by Roy et al. (2017) as a pre-post comparison in our meta-analysis.

All included studies used the DSM-IV based PTSD checklist (PCL) in either the civilian or specific versions (Weathers et al., 1994, 2001; Weathers and Ford, 1996) to assess PTSD symptoms. Four studies assessed depressive symptoms with the Patient Health Questionnaire Depression Scale (PHQ-9; Kroenke et al., 2001), one study used the PHQ-8 (Kroenke et al., 2009). Except for Roy et al. (2017), none of the studies assessed symptoms of anxiety. Therefore, we were not able to meta-analytically evaluate the effects of smartphone apps on anxiety symptoms.

The study by Owen et al. (2015) was excluded although PTSD symptoms were measured using the PCL-C via the app; the authors analyzed data from users who had downloaded and used the app between 2012 and 2014 (*N* = 3,462) and, thus, had aggregated over 12,449 sessions. Yet, sample characteristics during the time points of assessment were not readily available, making it unfeasible to calculate effect sizes for meta-analysis. Mean scores for the PCL-C changed in the study by Owen et al. (2015) from *M* = 57.2 (*SD* = 15.7) at the first session to *M* = 55.1 (*SD* = 16.6) at individual return sessions. Reger et al. (2015) subjected two active-duty military service members with a current diagnose of PTSD to 8 weeks of prolonged exposure treatment, half of the duration with the support of PE Coach and the other half without the app. Since the participants in this study were both receiving prolonged exposure treatment and Reger et al. (2015) used a crossover design, it was not possible to isolate the effects of self-administer app. Participants, however, indicated higher levels of satisfaction concerning the weeks in which they were supported by the app.

### Effects of Self-Management App-Based Interventions on PTSD Symptoms (Pre-post Comparisons)

**Figure 2:**
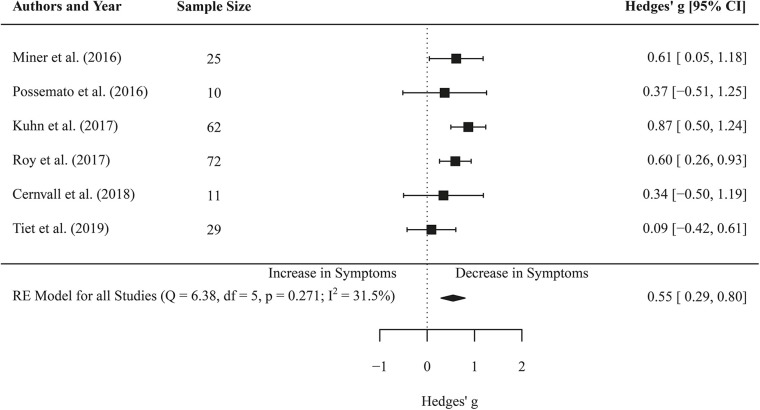
Forest plot of the standardized mean difference (Hedges' *g*) of the effect of self-management smartphone-based apps on PTSD symptoms (pre-post changes). A positive effect size indicates that the PTSD symptoms decreased at the post measurement.

Six effect sizes covering 209 participants were extracted to calculate the overall effect, operationalized in changes in PCL scores before and after the intervention. Meta-analysis concluded an effect of *g* = 0.55 (CI 0.29–0.80, *p* < 0.001) regarding the reduction in PTSD symptoms post intervention. Low heterogeneity between studies was found (*I*^2^ = 31.47, *Q*(5) = 6.38, *p* = 0.271). Meta-regression did not reveal a significant coefficient for the duration of the intervention on PTSD symptoms (*b* = −0.02, *SE* = 0.03, *p* = 0.622). See Figure 2 for forest plot.

### Effects of Self-Management App-Based Interventions on Depressive Symptoms (Pre-post Comparisons)

**Figure 3:**
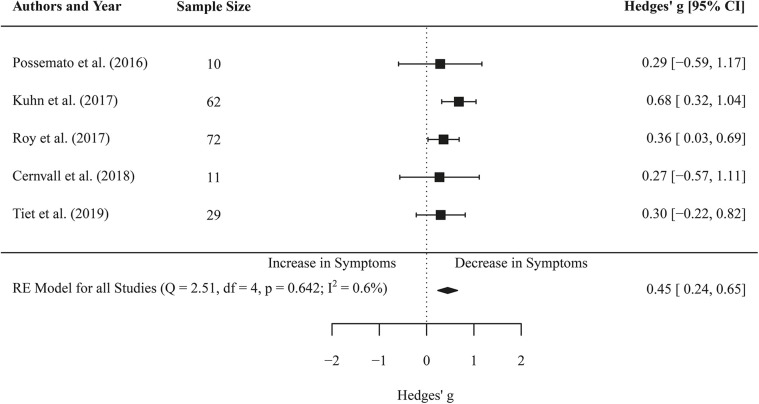
Forest plot of the standardized mean difference (Hedges' *g*) of the effect of self-management smartphone-based apps on depressive symptoms (pre-post changes). A positive effect size indicates that the depressive symptoms decreased at the post measurement.

Five effect sizes covered the changes in PHQ scores of 184 participants before and after the intervention. Meta-analysis revealed an effect of *g* = 0.45 (CI 0.24–0.65, *p* < 0.001). Low heterogeneity between studies was found for depressive symptoms (*I*^2^ = 0.58, *Q*(4) = 2.52, *p* = 0.642). Furthermore, meta-regression did not reveal a significant coefficient for the duration of the intervention on depressive symptoms (*b* = 0.01, *SE* = 0.03, *p* = 0.629). See Figure 3 for forest plot.

### Efficacy of Self-Management App-Based Interventions in Randomized Controlled Trials

Two studies (Miner et al., 2016; Kuhn et al., 2017; overall *N* = 169) compared app-based interventions to waitlist control-groups in randomized controlled trials. Meta-analysis of post-treatment scores in PTSD symptoms of these two studies resulted in no significant difference between app-based treatment and waitlist groups (*g* = 0.09 [CI −0.22–0.39], *p* = 0.574). No heterogeneity was found between the two studies (*I*^2^ = 0.00, *Q*(1) = 0.30, *p* = 0.584; results not shown). Interestingly, meta-analysis concluded an effect post treatment of *g* = 0.47 for PTSD symptom reduction in waiting list controls compared to an effect post treatment of *g* = 0.79 in the treatment groups. Studies were rated with moderate-high SOE.

### Publication Bias and Risk of Bias Assessment

**Figure 4:**
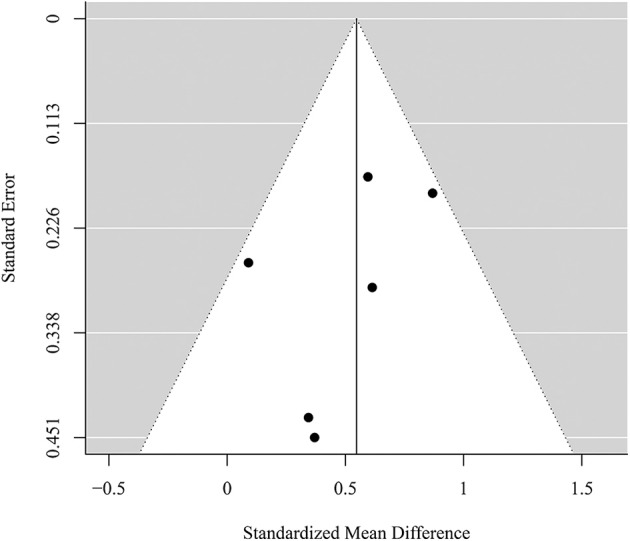
Funnel plot for the meta-analysis of the effect of self-management smartphone-based apps on PTSD symptoms.

**Figure 5:**
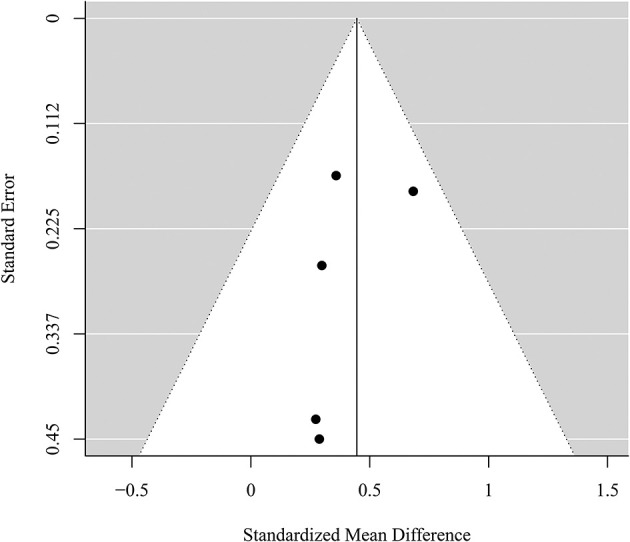
Funnel plot for the meta-analysis of the effect of self-management smartphone-based apps on depressive symptoms.

**Figure 6:**
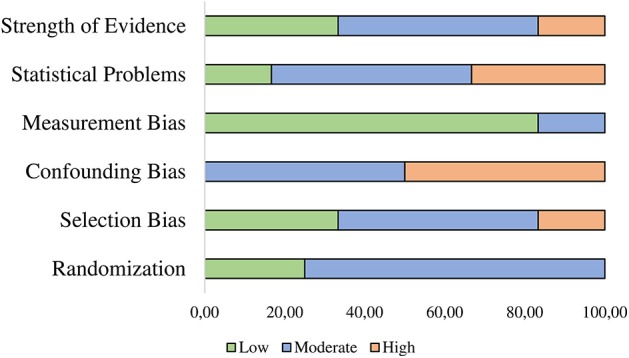
Graphical Representation of the Risk of Bias Assessment.

Visual inspection of the funnel plots (see Figures 4, 5) did not suggest a publication bias in the present meta-analysis. Results for Egger's regression for funnel plot asymmetry were not significant both for the analysis of PTSD symptoms (*z* = −1.09, *p* = 0.277) and the analysis of depressive symptoms (*z* = −0.67, *p* = 0.503). No adjustments were needed according to the trim-and-fill analysis (no studies added left of the summary effect) in both analyses. This suggests no indication for publication bias in the present meta-analysis. Studies are heterogeneous regarding strengths of evidence in overall quality of evidence assessment. Our review revealed that majority of studies showed high or moderate risk of bias as presented in Figure 6.

## Discussion

In light of the ever increasing, promising use of innovative technologies in the context of treatment, the current meta-analysis set out to systematically analyze the effect of self-management smartphone-based applications as a means of intervention in populations with PTSD. Six studies with an overall sample of 209 participants with both subthreshold and full PTSD who used one or more self-management applications as an intervention were included in the meta-analysis. PTSD as well as anxiety and depressive symptoms were used as outcomes.

In the overall sample, self-management smartphone-based applications showed a moderate effect size (*g* = 0.55) for the reduction of PTSD symptoms (assessed with the PCL) post treatment. In the two included RCTs with waitlist controls, however, no significant decrease in PTSD symptoms was found after the intervention (*g* = 0.09). Regarding depressive symptoms (assessed with the PHQ), the overall effect was *g* = 0.47, bordering on a moderate effect size. A separate analysis for depressive symptoms in RCTs was not possible, as they were not assessed in these trials. In addition, the effect of self-management apps on anxiety symptoms could not be analyzed as only one study (Roy et al., 2017) reported according scores. As anxiety symptoms are regarded a frequent comorbidity of PTSD (e.g., Ginzburg et al., 2010), it is crucial to systematically assess them in future controlled trials which evaluate the efficacy of PTSD interventions. This would allow for a more differentiated picture regarding the differential effect of according treatments on the reduction of anxiety.

Overall, the current results suggest that PTSD symptom severity is reduced while using self-management smartphone-based apps, yet, the factors to which these changes may be attributed remain unclear. The app-specific effect evaluated in the RCTs was not significant.

Unexpectedly, the results of our meta-analysis indicate that there is no difference between an app-based intervention and waitlist control conditions regarding PTSD symptom severity post treatment. This might be due to the small number of RCT studies (*n* = *2*) included in our analysis as well as to the high pre-post effect size of *g* = 0.47 for PTSD symptom reduction in the waitlist control group. A possible explanation for symptom reduction in the absence of treatment may be that the inclusion of a patient in a study often entails a beneficial shift in attentional focus. Even though no treatment is provided, the patient is still subject to repeated clinical assessments and receives support and information regarding his/her symptoms. Accordingly, Smith et al. (2007) found that patients improved significantly simply by monitoring their PTSD symptoms. This questions the efficacy of self-management applications and encourages further RCT research regarding smartphone-based apps, and furthermore, a deeper discussion of its usefulness as a stand-alone intervention. Moreover, the content of self-management apps used by most included studies was similar or even the same, limiting a possible generalization of the effects for other or future smartphone-based applications. Further research of content-based factors for treatment outcomes (e.g., level of interactivity, type of tasks such as relaxation tasks, self-monitoring tasks) would be beneficial for the field of smartphone-based therapy apps. Nevertheless, such research would be advantageous for all mobile applications in the context of psychological therapy. Hence, based on the current results, the conceptual integration of smartphone-based apps for self-management intervention in consisting therapies seems to be essential, as well as a further development to reach an exponentially higher efficacy with a combined treatment.

Moreover, findings suggest that depressive symptoms decrease during the use of smartphone-based apps. However, it was not possible to conduct additional meta-analysis to assess the app-specific effect on depression change in RCTs, as only one study (Kuhn et al., 2017) assessed depressive symptoms in a randomized control design. Kuhn et al. (2017) reported a reduction in depressive symptoms, yet—similarly to our meta-analysis of PTSD symptoms in RCTs—scores between the intervention and waitlist group did not differ at post treatment. Both the utilization and the prospect of being able to utilize apps appear to have a supportive, stress-buffering effect. This means, that the individual is protected against the detrimental consequences of stress over time through continuing support. Accordingly, a recent experimental study (Kothgassner et al., 2019a) succeeded in demonstrating a considerable stress buffering effect of virtually provided support compared with face-to-face support. Results indicate that acute stress regulation, negative emotions of shame and rumination—as essential markers for PTSD and depressive symptoms—improved when people received digitally mediated social support, yet this support was only effective in terms of stress buffering if participants thought it was provided by another person (via an avatar) and not by a computer (via an agent). Following this, it can be argued that the patients' assumption that they are being supported—either virtually or physically—by another human could be a crucial factor influencing the efficacy of innovative, interactive intervention apps and may limit the efficacy of apps providing only self-administered content without a supporting person.

Avatar-based technology facilitates several therapy approaches, as it can substitute face-to-face contact with a clinician. According to Rehm et al. (2016), two concepts exist of how to include avatars into therapy: On the one hand, the patient interacts with an avatar, this was used as an effective tool in Virtual Reality Exposure Therapy (e.g. Cárdenas and De La Rosa, 2012 for PTSD), and as the embodiment of a real clinician or a supporting tool for self-management technology (e.g., Pinto et al., 2016 for depressive symptoms). On the other hand, patients may represent themselves as a virtual avatar, either as representation of the self for assessment or to be involved in a therapy setting. As an avatar can be seen as digital representation of the self that may become part of a person's overall identity after a certain time (Bessière et al., 2007), it reflects a link to a person's personality, strengths, and impairments. Further, the matter of how individuals behave and interact via avatars can be used for assessment or therapeutic information. It has been shown that avatar preference of persons with traumatic events differs from persons without traumatic events and that there are differences between men and women with emotional or physical abuse regarding their choices of avatar characteristics. Women choose avatar characteristics to help others, while men tend to use aggressive features for their avatars (Kothgassner et al., 2020). Other studies already showed evidence for the effect of avatars as representations of the patient to assess PTSD symptoms through a computer-based avoidance task (Myers et al., 2016; e.g., Allen et al., 2017).

In sum, it is—at this point—difficult to deduce specific recommendation for future apps from the current results since all but one study (Roy et al., 2017) have used the same self-management app. The PTSD Coach entails four modules including psychoeducative elements (about the disorder itself as well as about treatment options and family relations), the option to track symptoms (i.e., in the form of repeated assessments of related thoughts and emotions), symptom management tasks (e.g., stress relief) as well as a feature for receiving support (e.g., in the event of crisis). In line with the idea of self-management, this app offers only limited interactivity with another person (e.g., psychologist, friends, peers etc.). Based on the consideration that social resources (e.g., involvement of significant others in the treatment process) and virtual social support (see above, Kothgassner et al., 2019a) may show particularly beneficial effects on treatment outcome (see Heaney and Israel, 2008), we may, with caution, suggest the inclusion of more social interactive elements in future apps, be it in the form of actual interactions (via chat, voice recordings, video etc.) or via a pre-programmed virtual human which implies the presence of another person. Being accompanied by an avatar throughout the online treatment process has proven beneficial in past studies (see Rehm et al., 2016 for a review). Further, this lack of knowledge regarding the design of smartphone-based therapy applications strengths the need for including therapy naïve and experienced patients in the development for future therapy applications.

In general, the effects of self-management smartphone-based intervention apps are smaller compared with the effects found by another meta-analysis on more established and evidence-based interventions for PTSD like prolonged exposure therapy (PE) (*g* = 1.08 for PTSD symptoms; see Powers et al., 2010). Similarly, studies in the field of child and adolescent trauma-focused cognitive behavioral therapy (tf-CBT) showed a higher effect (*d* = 0.88 for PTSD symptoms; see Goldbeck et al., 2016) compared to a waiting list control group. However, compared with another technology-mediated therapy approach—the Virtual Reality exposure therapy—larger effect sizes are found for PTSD and depression symptom reduction compared to waiting list controls in a recent quantitative review (*g* = 0.62; *g* = 0.50; see Kothgassner et al., 2019b). For the current results, the inclusion of subthreshold and full PTSD is a clear limitation, because self-management smartphone-apps may be helpful and supportive for people with experienced trauma and mild symptoms, but not for full PTSD. According to this, it is necessary to state that the inclusion of patients with subthreshold PTSD strongly limits comparability to other studies including only full PTSD patients for treatment. However, this is a major point for future original studies investigating smartphone-based interventions. In light of the present results, self-management smartphone-apps might be a supportive intervention, but not a stand-alone solution. Another shortcoming is the small number of studies included which made it impossible to evaluate the efficacy of self-management apps only via RCTs. Some studies were pilot trials and did not have randomized control groups, others did not have control conditions that would make comparisons feasible (e.g., treatment as usual or apps with clinician support). This was explicated by SOE ratings, showing only one study with high, yet two studies with low SOE. However, by including non-randomized studies and reporting an overall pre-post effect, we were able to analyze the efficacy of self-managed apps as a stand-alone intervention—-in a granulated manner—-with more confidence. Additionally, as the studies included in the meta-analysis predominantly used one specific smartphone-app for treatment it was not possible to compare different solutions and designs. This hinders generalization for all smartphone-app approaches treating PTSD symptoms.

Furthermore, third variables, which are not possible to control for, might have influenced the extracted effect sizes concerning PTSD and depressive symptoms. For instance, both the duration and the daily use of the applications seem to be vital for the method's success (Henson et al., 2019). Although we did not find a moderating effect of duration, it was not possible to test for the actual use of applications in the daily life due to a lack of consistent reporting. Only few authors assessed use of applications in self-report; here, individuals indicated that they used the mobile app between 2.27 (Kuhn et al., 2017) and 2.65 times a week (Miner et al., 2016). The interventions' duration ranged between fo0ur and 12 weeks in the included studies, and it did not explain heterogeneity neither in PTSD nor in depressive symptoms. Future studies should investigate the relation between frequency of usage and improvement of symptoms.

Standardizing treatment duration, frequency of usage, and comparing key outcomes to treatment-as-usual control groups in a randomized controlled design would certainly add to a better understanding of processes underlying the efficacy of smartphone-based intervention applications for PTSD for example by mediation analyses. Another open question pertains to the fact that, to date, it is unclear how patients with PTSD perceive health-related mobile apps in terms of usability and acceptability (Rodriguez-Paras et al., 2017). This, however, may be a crucial issue when it comes to patient compliance and adherence in the context of mobile health applications, in particular with regards to self-management but also regarding data protection and security concerns. Understanding these technologies and perceiving them as useful may be an essential prerequisite for an adequate usage by patients. Furthermore, the investigation of guided and unguided support via smartphone apps could be a future interest for research in PTSD treatment. Research synthesis already showed guidance as a beneficial feature in Internet- and mobile-based interventions and reveals that clinical qualification of the person providing guidance is surprisingly of minor importance (Baumeister et al., 2014). Furthermore, first results concerning Internet- and mobile-based interventions used as supportive, adjunct tools in face-to-face therapy (blended care) seem promising (e.g., in the context of depression, Berger et al., 2018).

## Conclusion

The current meta-analysis found small-to-moderate pre-post effect sizes for the reduction of PTSD and depressive symptoms in an overall sample of 209 participants. Even though effects are smaller than those of typical evidence-based interventions and therapies for PTSD (Powers et al., 2010), smartphone-based apps—due to their reach and availability—have a considerable potential to become vital parts of treatment strategies and interventions for communities and military populations suffering from subthreshold PTSD. In particular, the option of assessing health data on a day-to-day basis and in an ecologically valid fashion would not only allow for pinpointed assessments of key symptoms in future. It would also add to more customized technology-based interventions with an improved interaction between patient needs and clinician resources.

The results of our study imply that a self-managed smartphone-based app is not superior to waitlist control. It might therefore not be recommended to use these tools as stand-alone interventions. Following recent research, the social component seems to be important in basic computer mediated as well as in more complex virtual social interactions. According to this, it is safe to assume that a professional social entity is needed for a significant impact on symptomatology (e.g., Kothgassner et al., 2019a), but further smartphone-based apps have the potential to enrich traditional therapy protocols. Currently, there is a definitive lack of research on combined treatments (traditional face-to-face therapy including mobile app interventions) in the field of PTSD treatment. Evaluating the benefits of such blended care approaches during PTSD therapy as well as in the context of ambulatory recovery seems to be a particularly fruitful field for future research.

## Data Availability Statement

All datasets generated for this study are included in the article/supplementary material.

## Author Contributions

AG and OK wrote the first draft of the manuscript. JK and OK conducted the literature search and coded the studies. AG prepared the statistical procedures and analyzed the data. AF, JK, and TP contributed extensively to the first draft. All authors have approved the final manuscript.

### Conflict of Interest

The authors declare that the research was conducted in the absence of any commercial or financial relationships that could be construed as a potential conflict of interest. The reviewer SS declared a shared affiliation with no collaborations with one of the Authors AG to the handling editor at the time of review.
