# Supplementary material for: Efficacy of Self-Management Smartphone-Based Apps for Post-traumatic Stress Disorder Symptoms: A Systematic Review and Meta-Analysis
Source: Front Neurosci. 2020 Jan 24;14:3. doi: 10.3389/fnins.2020.00003 (PMC6992648; doi:10.3389/fnins.2020.00003)
Supplement: Supplementary file 1 [file Table_1.docx]

**Supplementary Material**

Supplementary Table 1 shows Risk of Bias Assessments–predefined criteria for rating. In Table 2, we present the six studies’ assessment of quality sorted by design (Randomized Controlled Trials; RCTs, pre-post design), and in Supplementary Table 3, the PRISMA checklist.

**Supplementary Table 1: Risk of Bias Assessment – Categories for rating (based on Viswanathan et al., 2018)**

| **Randomization** |  |
| --- | --- |
| Method of Randomization* | **Low**: Computer generated random allocation  **Moderate**: Other methods identified to randomize  **High**: Inadequate method of randomization (e.g., alternating), randomization approach cannot be determined or did not randomize |
| **Selection Bias** |  |
| Attrition Bias | **Low:** Low attrition or low differential loss  **Moderate:** Moderate attrition (20-39%) or moderate differential loss (5-29%)  **High:** High Attrition (≥ 40%) or high differential loss (≥ 30%), or cannot be determined |
| Creation of Comparable  Groups* | **Low:** No significant baseline differences among groups regarding primary outcome measures and inclusion/exclusion criteria  **Moderate:** Few baseline difference among groups  **High**: Multiple differences among groups, or does not state any baseline comparisons between groups |
| **Confounding Bias** |  |
| Control of confounding | **Low:** Addressed through study design (e.g., RCT) and/or statistical analysis  **Moderate:** Attempt made to control confounding, but doesn’t address all relevant confounders  **High:** No attempt to control confounders |
| **Measurement Bias** |  |
| Outcome Measurement | **Low:** Existing and validated measure used for PTSD symptom severity; and at least one secondary outcome measures (e.g. depressive symptoms, anxiety symptoms, coping behavior, quality of life)  **Moderate:** Validated measurement for PTSD symptom severity  **High:** Used originally developed instrument/items without validation |
| **Statistical Problems**** | **Low:** Power-calculation, sufficient participants, reporting p-values and effect size estimates, using appropriate statistical methods  **Moderate:** Reporting p-values and effect size estimates, appropriate statistical methods  **High:** Small group sizes, insufficient data, or inappropriate statistical methods used |
| **Grades of Overall Strength of Evidence according to Owens et al. (2010)** | **High:** High confidence that the evidence reflects the true effect. Further research is very unlikely to change our confidence in the estimate of effect.  **Moderate:** Moderate confidence that the evidence reflects the true effect. Further research may change our confidence in the estimate of the effect and may change the estimate.  **Low: L**ow confidence that the evidence reflects the true effect. Further research is likely to change our confidence in the estimate of the effect and is likely to change the estimate.  **Insufficient:** Evidence either is unavailable or does not permit estimation of an effect. |

* Indicator used only for RCTs

** Only Kuhn et al. (2017), but none of the other studies reported power calculations to determine if they had sufficient samples sizes to assess effectiveness of their interventions

**Supplementary Table 2: Risk of Bias Assessments**

|  | **Study** | **Randomization** | **Selection Bias** | **Confounding Bias** | **Measurement Bias** | **Statistical Problems** | **Strength of Evidence** |
| --- | --- | --- | --- | --- | --- | --- | --- |
| **RCTs** | Kuhn et al. (2017) | Low | Low | Moderate | Low | Low | High |
|  | Miner et al. (2016) | Moderate | Low | Moderate | Moderate | Moderate | Moderate |
|  | Possemato et al. (2016) | Moderate | Moderate | High | Low | High | Low |
|  | Roy et al. (2017) | Moderate | Moderate | Moderate | Low | Moderate | Moderate |
|  |  |  |  |  |  |  |  |
| **Pre-Post Trials** | Cernvall et al. (2018) | N. A. | High | High | Low | High | Low |
|  | Tiet et al. (2019) | N. A. | Moderate | High | Low | Moderate | Moderate |

**Supplementary Table 3. PRISMA checklist**

| **Section/topic** | **#** | **Checklist item** | **Reported on page #** |
| --- | --- | --- | --- |
| **TITLE** | | |  |
| Title | 1 | Identify the report as a systematic review, meta-analysis, or both. | 1 |
| **ABSTRACT** | | |  |
| Structured summary | 2 | Provide a structured summary including, as applicable: background; objectives; data sources; study eligibility criteria, participants, and interventions; study appraisal and synthesis methods; results; limitations; conclusions and implications of key findings; systematic review registration number. | 2 |
| **INTRODUCTION** | | |  |
| Rationale | 3 | Describe the rationale for the review in the context of what is already known. | 3–5 |
| Objectives | 4 | Provide an explicit statement of questions being addressed with reference to participants, interventions, comparisons, outcomes, and study design (PICOS). | 5 |
| **METHODS** | | |  |
| Protocol and registration | 5 | Indicate if a review protocol exists, if and where it can be accessed (e.g., Web address), and, if available, provide registration information including registration number. | NA |
| Eligibility criteria | 6 | Specify study characteristics (e.g., PICOS, length of follow-up) and report characteristics (e.g., years considered, language, publication status) used as criteria for eligibility, giving rationale. | 5 |
| Information sources | 7 | Describe all information sources (e.g., databases with dates of coverage, contact with study authors to identify additional studies) in the search and date last searched. | 5 |
| Search | 8 | Present full electronic search strategy for at least one database, including any limits used, such that it could be repeated. | 5 |
| Study selection | 9 | State the process for selecting studies (i.e., screening, eligibility, included in systematic review, and, if applicable, included in the meta-analysis). | 5 |
| Data collection process | 10 | Describe method of data extraction from reports (e.g., piloted forms, independently, in duplicate) and any processes for obtaining and confirming data from investigators. | 5 |
| Data items | 11 | List and define all variables for which data were sought (e.g., PICOS, funding sources) and any assumptions and simplifications made. | 5 |
| Risk of bias in individual studies | 12 | Describe methods used for assessing risk of bias of individual studies (including specification of whether this was done at the study or outcome level), and how this information is to be used in any data synthesis. | 5–6 |
| Summary measures | 13 | State the principal summary measures (e.g., risk ratio, difference in means). | 5–6 |
| Synthesis of results | 14 | Describe the methods of handling data and combining results of studies, if done, including measures of consistency (e.g., I^2^) for each meta-analysis. | 5–6 |

| **Section/topic** | **#** | **Checklist item** | **Reported on page #** |
| --- | --- | --- | --- |
| Risk of bias across studies | 15 | Specify any assessment of risk of bias that may affect the cumulative evidence (e.g., publication bias, selective reporting within studies). | 6 |
| Additional analyses | 16 | Describe methods of additional analyses (e.g., sensitivity or subgroup analyses, meta-regression), if done, indicating which were pre-specified. | 5–6 |
| **RESULTS** | | |  |
| Study selection | 17 | Give numbers of studies screened, assessed for eligibility, and included in the review, with reasons for exclusions at each stage, ideally with a flow diagram. | 6–7 |
| Study characteristics | 18 | For each study, present characteristics for which data were extracted (e.g., study size, PICOS, follow-up period) and provide the citations. | 6–7, Table 1 |
| Risk of bias within studies | 19 | Present data on risk of bias of each study and, if available, any outcome level assessment (see item 12). | 7, Supplementary Material |
| Results of individual studies | 20 | For all outcomes considered (benefits or harms), present, for each study: (a) simple summary data for each intervention group (b) effect estimates and confidence intervals, ideally with a forest plot. | Figures 2–3 |
| Synthesis of results | 21 | Present results of each meta-analysis done, including confidence intervals and measures of consistency. | 6–8 |
| Risk of bias across studies | 22 | Present results of any assessment of risk of bias across studies (see Item 15). | 8 |
| Additional analysis | 23 | Give results of additional analyses, if done (e.g., sensitivity or subgroup analyses, meta-regression [see Item 16]). | 7–8 |
| **DISCUSSION** | | |  |
| Summary of evidence | 24 | Summarize the main findings including the strength of evidence for each main outcome; consider their relevance to key groups (e.g., healthcare providers, users, and policy makers). | 8–9 |
| Limitations | 25 | Discuss limitations at study and outcome level (e.g., risk of bias), and at review-level (e.g., incomplete retrieval of identified research, reporting bias). | 9–11 |
| Conclusions | 26 | Provide a general interpretation of the results in the context of other evidence, and implications for future research. | 11–12 |
| **FUNDING** | | |  |
| Funding | 27 | Describe sources of funding for the systematic review and other support (e.g., supply of data); role of funders for the systematic review. | 12 |

**References**

Viswanathan, M., Patnode, C. D., Berkman, N. D., Bass, E. B., Chang, S., Hartling, L., ... & Kane, R. L. (2018). Recommendations for assessing the risk of bias in systematic reviews of health-care interventions. *Journal of Clinical Epidemiology, 97*, 26–34. doi:10.1016/j.jclinepi.2017.12.004

Owens, D. K., Lohr, K. N., Atkins, D., Treadwell, J. R., Reston, J. T., Bass, E. B., ... & Helfand, M. (2010). AHRQ series paper 5: grading the strength of a body of evidence when comparing medical interventions—Agency for Healthcare Research and Quality and the Effective Health-Care Program. *Journal of Clinical Epidemiology*, *63*, 513–523. doi:10.1016/j.jclinepi.2009.03.009
